# MG53 promotes corneal wound healing and mitigates fibrotic remodeling in rodents

**DOI:** 10.1038/s42003-019-0316-7

**Published:** 2019-02-20

**Authors:** Heather L. Chandler, Tao Tan, Chunlin Yang, Anne J. Gemensky-Metzler, Rita F. Wehrman, Qiwei Jiang, Cornelia M. W. Peterson, Bingchuan Geng, Xinyu Zhou, Qiang Wang, Denis Kaili, T. M. Ayodele Adesanya, Frank Yi, Hua Zhu, Jianjie Ma

**Affiliations:** 1 College of Optometry, The Ohio State University, Columbus, USA; 2 College of Veterinary Medicine, The Ohio State University, Columbus, USA; 3 Department of Surgery, The Ohio State University Wexner Medical Center, Columbus, USA; 4 TRIM-edicine, Columbus, USA

## Abstract

The cornea plays an important role in transmitting light and providing protection to the eye, but is susceptible to injury and infection. Standard treatments for corneal wounds include topical lubricants, antibiotics, bandage contact lens, and surgery. However, these measures are often ineffective. Here we show that MG53, a protein with an essential role in cell membrane repair, contributes to the corneal injury-repair process. Native MG53 is present in the corneal epithelia, tear film, and aqueous humor, suggesting its potential function in corneal homeostasis. Knockout of MG53 in mice causes impaired healing and regenerative capacity following injury. Exogenous recombinant human MG53 (rhMG53) protein protects the corneal epithelia against mechanical injury and enhances healing by promoting migration of corneal fibroblasts. Using in vivo alkaline-induced injury to the rat cornea, we show that rhMG53 promotes re-epithelialization and reduces post-injury fibrosis and vascularization. Finally, we show that rhMG53 modulates TGF-β-mediated fibrotic remodeling associated with corneal injury. Overall, our data support the bi-functional role of MG53 in facilitating corneal healing and maintaining corneal transparency by reducing fibrosis and vascularization associated with corneal injuries.

## Abstract (web summary)

Heather Chandler, Tao Tan, Chunlin Yang et al. find that the cell membrane repair protein MG53 plays a key role in repairing cornea injury. Using mouse and rat models, they show that recombinant human MG53 protects the cornea against injury and enhances healing.

## Introduction

The cornea is the eye’s outermost layer that plays an important role in transmitting light and providing protection to the intraocular components of the eye. Due to its exposure to the external environment, the cornea is susceptible to injury and infection. Because the cornea is densely innervated, sustained corneal wounds can be painful and delays in repair can increase the risk of corneal scarring and vision loss. The standard treatment of complicated corneal wounds includes maximizing topical lubricants, minimizing evaporative tear loss, using topical antibiotics, protecting the corneal surface with a bandage contact lens, and undergoing surgery^1^. However, even in combination, these measures are often ineffective^1–3^. Although treatment of corneal wounds with specific growth factors and autologous serum may have promise^4–7^, to date, only one biologic (recombinant human neuron growth factor, rhNGF, cenegermin) has been approved for clinical application for promoting epithelial healing^8^. This leaves many clinicians with limited treatment options when dealing with a complicated corneal ulcer and as such, there is an unmet need for therapies to treat corneal wounds. The knowledge gap in understanding the molecular mechanisms associated with repair of corneal injuries is an impediment in the development of effective therapies to treat corneal wounds.

Corneal wound healing is a complex and coordinated physiological process, involving repair to the epithelial layer, migration of viable epithelial cells and fibroblasts for wound closure, and stimulation of cellular proliferation for tissue regeneration^9^. Prevention of excessive myofibroblast activation and vascular ingrowth is also imperative to avoid fibrosis and angiogenesis, which can compromise the transparency of the cornea^9^. Thus, an approach that can functionally target multiple steps in corneal wound healing may have the potential to improve healing outcomes, leading to novel therapeutic options.

Following injury, cell membrane repair is an important aspect of physiology and inadequate membrane repair contributes to the pathophysiology of several human diseases, including ocular dysfunction^10,11^. MG53 is a member of the TRIM protein family that has an essential role in cell membrane repair^12–15^. MG53 acts as a sensor of oxidation to oligomerize and recruit intracellular vesicles to the injury site allowing for membrane patch formation^12^. Genetic ablation of MG53 results in defective membrane repair and skeletal and cardiac muscle derived from *mg53−/−* mice have been shown to be more susceptible to stress-induced injuries^16,17^.

While MG53 is an intracellular protein, physiological activity or injury to skeletal or cardiac muscle can lead to secretion of MG53 into the systemic circulation^17–19^. As such, serum levels of MG53 can serve as paracrine factors for protection against stress-induced tissue injuries, especially for tissues with low expression of endogenous MG53^17–20^. Using in vivo animal models, we have previously shown that intravenous delivery of the recombinant human MG53 (rhMG53) protein could repair membrane damage to muscle and non-muscle cells, and ameliorated the pathology associated with muscular dystrophy^19^, myocardial infarction^18^, acute lung injury^21^, and acute kidney injury^22^.

In the present study, we investigate the physiological function of MG53 in preserving the integrity of the cornea following injury. We also provide evidence that MG53 is present in the corneal epithelia, tear film, and aqueous humor. Thus, therapeutic approaches involving rhMG53 protein are unlikely to invoke ocular inflammatory responses. Our findings reveal a bi-functional role for MG53 in facilitating rapid injury-repair of corneal wounds and reducing fibrotic vascularization associated with corneal injuries. Our data support the therapeutic value for targeting MG53 function to treat ocular injuries and prevent fibrosis associated with corneal diseases.

## Results

### MG53 is expressed in cornea and repairs corneal epithelial cell injury

**Fig. 1:**
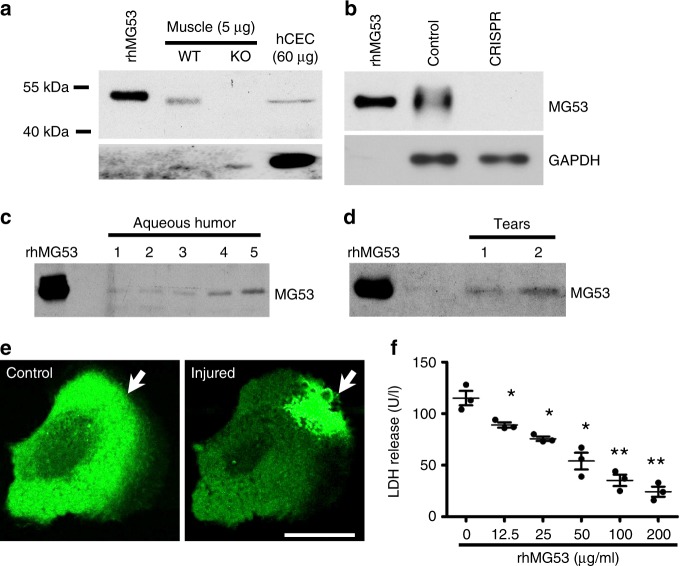
MG53 participates in membrane damage repair in hCEC. **a** Western blot analysis shows MG53 in hCEC (60 µg); rhMG53 (0.2 ng) and skeletal muscle lysates from wildtype mice (5 µg) were used as positive controls. Muscle from *mg53−/−* was used as a negative control. **b** CRISPR/Cas 9 mediated knockout of MG53 in hCEC was performed and confirmed by Western blot. Endogenous MG53 is present in both the canine aqueous humor (**c**) and **d** human tear film (20 μl of aqueous humor or tear sample was loaded to each lane. Each lane represented human and canine samples derived from different individuals), rhMG53 (0.4 ng) was loaded for quantification purpose. **e** GFP-MG53 expressed in hCEC, translocates to mechanical injury sites following microelectrode penetration (white arrow). Scale bar: 20 μm. **f** rhMG53 treatment prevents LDH release following glass bead damage. *p < 0.05; **p < 0.01

Using western blotting, we detected native MG53 protein in human telomerase-immortalized corneal epithelial cells (hCEC), although the level in hCEC was lower than that in muscle tissue (Fig. 1a). The identity of the MG53 protein in hCEC was confirmed using CRISPR/Cas-9 gene knockout. As shown in Fig. 1b, co-transfection of hCEC with Cas9 and specific gRNA targeting human MG53 led to near complete knockout of MG53 expression, when compared with untransfected hCEC.

Previously, we showed that MG53 is present in serum derived from the blood of mice, rats, and humans^17,18^. Using canine aqueous humor, native MG53 protein was detected (Fig. 1c). Semi-quantitative western blot analysis showed approximately ~0.7 ng mL^−1^ of MG53 protein in the aqueous humor. Moreover, tears derived from healthy human volunteers also contained low levels of MG53 protein, at ~3.1 ng mL^−1^ (Fig. 1d). These findings support the potential function of MG53 in maintaining ocular tissue integrity, and further suggest that application of exogenous MG53 protein is unlikely to cause potential toxicological and immunological responses in the eye.

To evaluate the function of MG53 in membrane repair following mechanical injury, hCECs were transfected with GFP-MG53. As shown in Fig. 1e, GFP-MG53 expressed in hCEC is localized to the cytosol and intracellular vesicles, a subcellular distribution that is similar to those observed in striated muscle^12^ and other non-muscle cell types^19^. In response to injury caused by penetration of a micro-electrode into the membrane, rapid translocation of GFP-MG53 labeled intracellular vesicles towards the injury site was observed. This GFP-MG53 translocation to membrane injury sites in hCEC is similar to that observed in muscle and non-muscle cells^12,19^.

Previous studies from our laboratory have shown that rhMG53 protein can protect various cell types against cell membrane disruption when applied to the extracellular solution^19,21^. To determine if exogenous rhMG53 can protect hCEC from mechanical injury, micro glass-beads were used to induce injury to the cells following our published procedure^14,19^. As shown in Fig. 1f, application of rhMG53 in culture medium protected hCEC viability in a dose-dependent manner, suggesting a potential application for rhMG53 in corneal wound healing.

### *mg53−/−* corneas show poor healing outcomes following injury

**Fig. 2:**
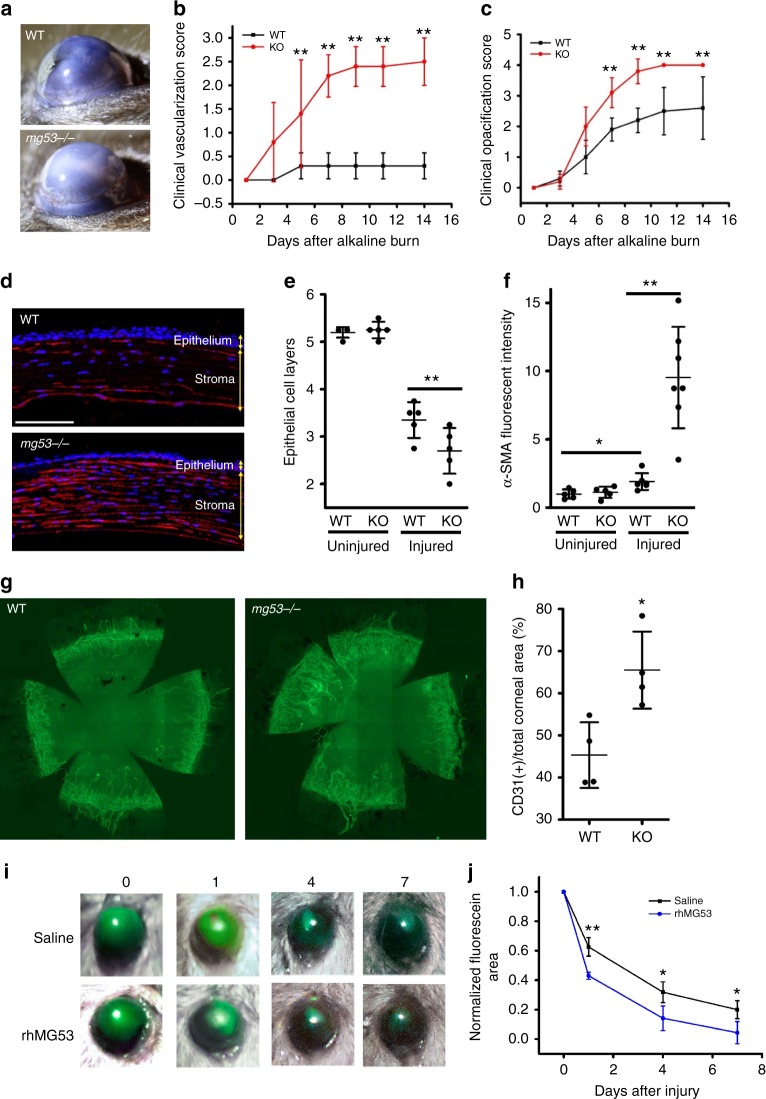
*mg53−/−* corneas demonstrate poor healing outcomes following alkali induced injury. **a** At day 7 post alkaline injury, cornea derived from the *mg53−/−* mice had increased vascularization than corneas derived from the wildtype littermate. **b** The *mg53−/−* cornea displayed elevated vascularization (**a** right panel, **b**) and opacification scores (**a** right panel, **c**). **d** Immunofluorescent confocal images revealed that injured *mg53−/−* corneas have significantly fewer epithelial cell layers (quantified in **e**) and more fibrosis (as demonstrated by α-SMA staining red in **d** and quantified in **f**) than wildtype corneas. Scale bar: 100 μm. **g** CD31 staining of flat mount corneas show that *mg53−/−* corneas have more vascularization than wildtype corneas (quantified in **h**). (*n* = 4 per group). *p < 0.05; **p < 0.01. **i** Representative image of fluorescein uptake showed that rhMG53 treatment improved re-epithelialization in *mg53−/−* corneas following alkali injury as compared to saline treatment as control (quantified in **j**). (*n* = 3 per group)

To elucidate the physiological role of MG53 in corneal wound healing, an alkaline eye injury model was applied to the *mg53−/−* and wildtype littermate mice, following published protocols^23^. Representative images, shown in Fig. 2a taken at day 7 post-alkaline injury, illustrated that the *mg53−/−* corneas had increased vascularization and opacification compared with corneas derived from wildtype mice. In our double-masked clinical analyses, the *mg53−/−* corneas consistently displayed elevated vascularization (Fig. 2b) and opacification (Fig. 2c) during the 14-days following alkaline injury.

At 14-days post-alkaline injury, globes were fixed either for horizontal sectioning or flat mount staining for histological evaluation. As shown in Fig. 2d, e, the injured *mg53−/−* corneas had significantly fewer epithelial layers than that of wildtype (3.4 ± 0.6 in wildtype vs. 2.7 ± 0.6 in *mg53−/−*, *p* < 0.01). Immunohistochemical staining with alpha-smooth muscle actin (α-SMA), a specific fibrosis marker, revealed that injured *mg53−/−* corneas had nearly a five-fold increase in α-SMA expression compared to controls (Fig 2d, f). When flat mount corneas were stained with antibodies against CD31 (a blood vessel marker, Fig. 2g), we found that the *mg53−/−* corneas had increased vascular encroachment into the axial cornea (CD31 positive area increased by 40% in *mg53−/−* corneas as compared to that in wildtype corneas, *p* < 0.05), further suggesting pronounced angiogenesis in *mg53−/−* corneas following injury. In order to test whether the observed defects in wound healing was due to loss of MG53, we used rhMG53 to treat alkaline injured *mg53−/−* corneas. As shown in Fig 2i, j, treatment of rhMG53 (100 ng mL^−1^) significantly improved re-epithelialization at 1, 4, and 7 days after alkaline injury as compared to those treated with saline as a control.

Overall, these in vivo studies indicate that *mg53−/−* corneas show poorer wound healing outcomes following alkaline injury as compared to wildtype littermates, supporting a critical role of MG53 in corneal physiology. Furthermore, treatment of rhMG53 could significantly improve healing capacity of *mg53−/−* corneas.

### rhMG53 improves healing and reduces fibrosis/vascularization

**Fig. 3:**
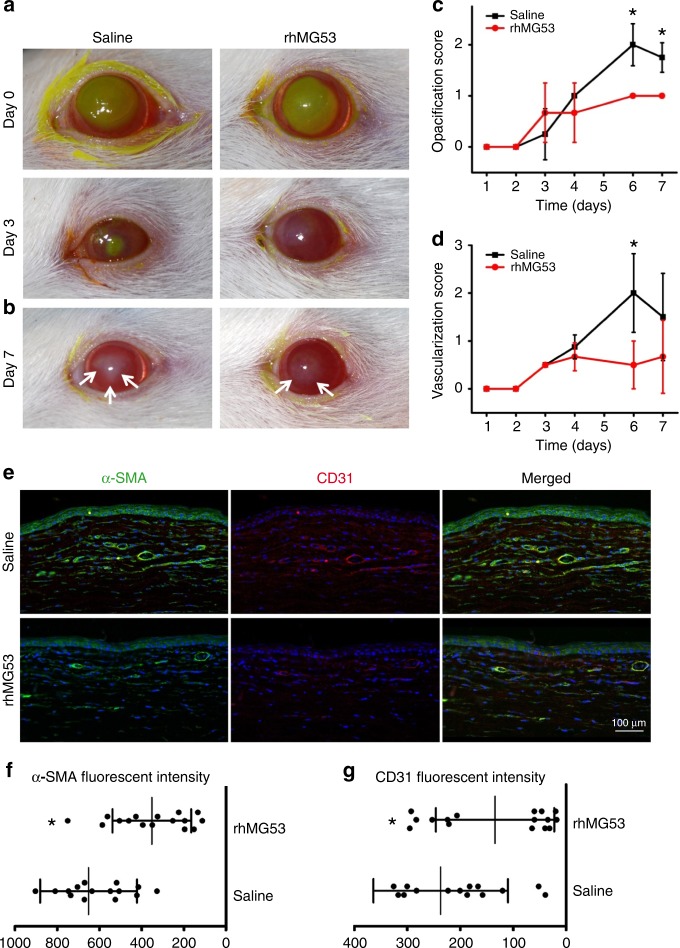
rhMG53 treatment improves corneal wound healing outcomes. **a** Rats receiving rhMG53 treatment exhibited reduced corneal fluorescein uptake at day 3 post alkaline-injury. Re-epithelization was defined by the day in which fluorescein was excluded. On average, rhMG53 treatment reduced re-epithelization from 4.0 ± 1.4 (saline) to 2.8 ± 0.8 days (+rhMG53). **b** On day 7 post alkaline injury, corneas that received topical rhMG53 showed reduced haze area and density, as outlined by the white arrows, as compared with those receiving saline alone. The clinical scores for opacification (**c**), and vascularization (**d**), were quantified at different days post alkaline-injury. rhMG53 treatment group (red) showed reduced fibrosis and vascularization. **e** IHC staining showed CD31 (vascularization marker) (left panels) and α-SMA (fibrosis marker) (right panels) staining were noted in both the superficial and deep axial cornea of control eyes. By comparison, stromal changes in the rhMG53 treated corneas were mitigated (bottom panels). Statistical analysis showed significant reduction in α-SMA (**f**) and CD31 (**g**) intensity in rats that received rhMG53 treatment (*p* < 0.05)

Based on the above findings, we tested the efficacy of rhMG53 protein in protecting corneal integrity using a rat alkaline-induced corneal injury model (Fig. 3). A total of 16 rats were used, with 8 receiving saline as control, and 8 receiving rhMG53 (100 ng mL^−1^), topically administered twice daily for 7 days. The clinical re-epithelialization, fibrotic, and vascularization scores were determined by a veterinary ophthalmologist.

Exclusion of fluorescein dye was used as an indicator for re-epithelization following injury (Fig. 3a). Injured rats that received rhMG53 treatment excluded fluorescein earlier (right), when compared with those receiving saline alone. At day 7 post-alkaline injury, reduced fibrosis was also observed in rats that received rhMG53 treatment (Fig. 3b). Clinical evaluations by an ophthalmologist masked to the treatment found that rhMG53-treated rats had significantly reduced opacification starting at day 6, post alkaline-injury (*p* < 0.05) (Fig. 3c). During the corneal healing process, the rhMG53 treated animals consistently show reduced vascularization scores with a significant difference observed on day 6 (*p* < 0.05) (Fig. 3d).

Histology was performed on all corneas at the termination of the study. Evaluation of CD31 demonstrated the reduction of superficial and deep vessels in corneal sections derived from rats treated with rhMG53, compared to those receiving saline as control (Fig. 3e). Expression of α-SMA and CD31 was also substantially reduced in MG53-treated corneas when compared to saline-treated corneas, demonstrating reduced activation of myofibroblasts (Fig. 3f, g).

Overall, the in vivo corneal injury studies support the therapeutic value for rhMG53 in improvement of corneal healing following alkaline injury.

### MG53 promotes in vitro corneal fibroblast migration

**Fig. 4:**
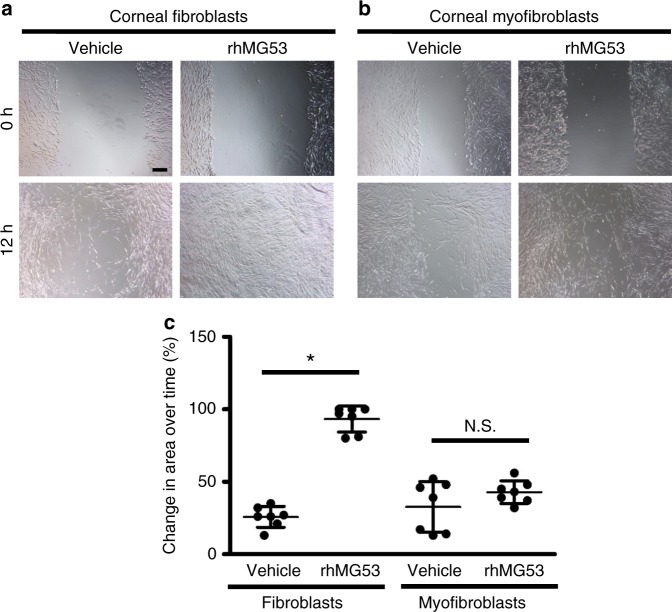
MG53 enhances migration of corneal fibroblasts but not myofibroblasts. Following scratch wound, application of rhMG53 accelerated migration of corneal **a** fibroblasts but not **b** myofibroblasts. **c** Change in area over time for both fibroblasts and myofibroblasts at 12 h after treatment was quantified. *n* = 7 independent experiments. *p < 0.01. Scale bar: 100 µm

Toward understanding the mechanisms that underlie MG53’s function to improve healing following corneal injury, we used an in vitro scratch healing model. It has been shown that migration of fibroblasts are important for corneal wound healing process^24,25^. Vehicle-treated cultured corneal fibroblasts and myofibroblasts underwent the expected migration and proliferation in response to scratch wounding (Fig. 4). Addition of rhMG53 to the culture media improved the healing process by accelerating the migration of fibroblasts (Fig. 4a). Interestingly, there was no significant increase in corneal myofibroblast wound healing in the presence of rhMG53 (Fig. 4b, summarized in Fig. 4c).

This data suggests that there is possible cell specificity to MG53’s effects, such that myofibroblasts do not exhibit a robust response following exposure to rhMG53. Such cell specificity may play a relevant role in corneal fibrosis and the mechanism of this phenomenon requires further investigation.

### MG53 prevents fibrosis through inhibition of TGF-β signaling

Extensive evidence suggests that TGF-β functions as an important cytokine in the pathogenesis of fibrotic corneal diseases^26–28^. As we observed that MG53 suppressed fibrosis during corneal healing, we asked whether MG53 played a role in cross-talk with TGF-β signaling.

**Fig. 5:**
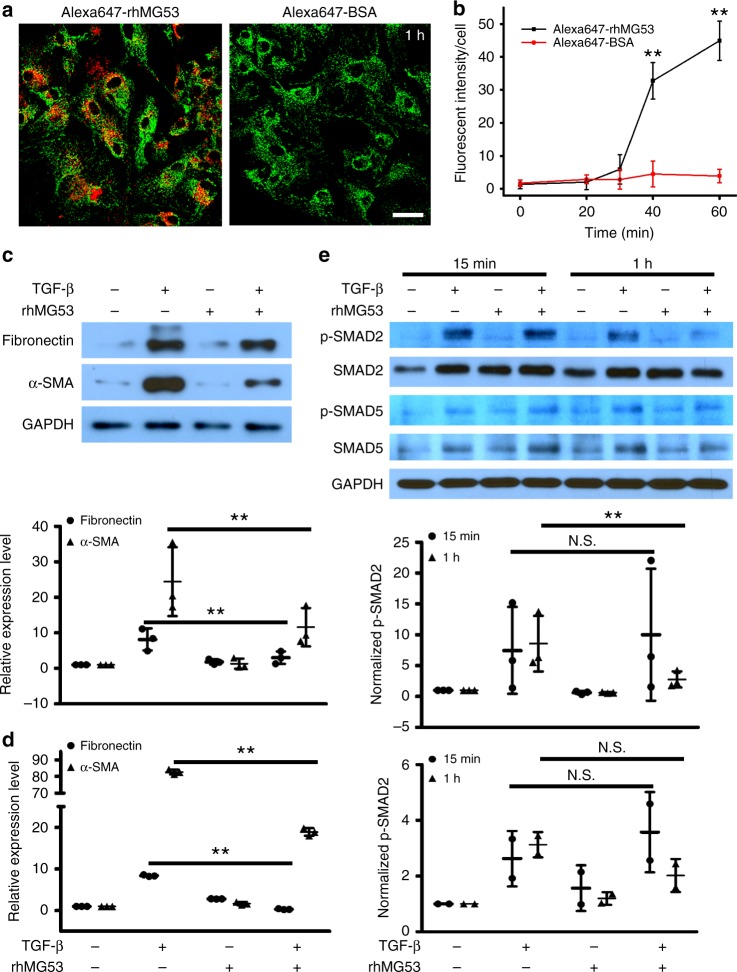
rhMG53 enters fibroblasts and prevents fibrotic remodeling through inhibiting TGF-β signaling. **a** Live cell imaging of corneal fibroblasts after 1 h incubation with Alexa647-rhMG53 (left) and Alexa647-BSA (right). Mitotracker green was used for counter staining to show cell shape. **b** Dynamics of rhMG53 entry was quantified. **c** Western blotting and **d** real-time RT-PCR showed that rhMG53 treatment significantly reduced TGF-β induced expression of fibronectin and α-SMA. **e** After 1 h incubation, TGF-β mediated activation of Smad2 was inhibited by rhMG53 (filled). With 15 min incubation, there was no effect of rhMG53 on TGF-β-induced activation of p-Smad2 (open). rhMG53 did not affect the level of p-Smad5 either with 15 min or 1 h incubation (bottom panel). *n* = 3 independent experiments. **p < 0.01. NS, no significant difference

Based on western blotting, we did not detect endogenous MG53 protein in primary cultured fibroblasts derived from the canine cornea. We thus performed live cell imaging to determine if rhMG53, when added to the extracellular space, could enter the corneal fibroblasts. For this purpose, rhMG53 was conjugated with Alexa647 to allow for live cell imaging under confocal microscopy. As shown in Fig. 5a, Alexa647-rhMG53 quickly entered the fibroblasts, whereas Alexa647-BSA (as control) failed to penetrate the cells. We further used live cell imaging to determine the time course of rhMG53 uptake and found that the entry of rhMG53 occurred 30 min after it was added to the extracellular space (Fig. 5b).

Based on the above measurement, we conducted biochemical assays to investigate the effect of rhMG53 treatment on TGF-β-mediated fibrotic signaling in corneal fibroblasts. Serum-starved canine fibroblasts were treated with TGF-β, and expression of fibronectin and α-SMA, as fibrotic markers, were quantified. As seen in Fig. 5c, d, while TGF-β promoted expression of fibrotic factors, co-treatment of rhMG53 significantly reduced the TGF-β-induced effects.

To differentiate the extracellular and intracellular actions of rhMG53, we treated fibroblasts with TGF-β and/or rhMG53 for either 15 min or 1 h. By differentiating the actions of rhMG53 at different time points, we hoped to determine the potential targets of rhMG53 in the TGF-β pathway. We detected the activation status of phosphorylated Smad2 (p-Smad2) as an indicator of the canonical TGF-β pathway, and the activation status of p-Smad5 as a factor of the BMP pathway, a non-TGF-β pathway which has also been shown to play a role in corneal fibrosis (Fig. 5e). Elevated levels of p-Smad2 were detected in cells following treatment with TGF-β. Notably, fibroblasts that received co-treatment of TGF-β and rhMG53 had reduced levels of p-Smad2, but not p-Smad5, indicating the action of rhMG53 was TGF-β specific. More importantly, inhibition of rhMG53 on p-Smad2 happened at 1 h of treatment but not following 15 min of treatment. Given that entry of rhMG53 into fibroblasts happens 30 min post-administration (Fig. 5b), our data indicates that it is the intracellular action of rhMG53 that interferes with TGF-β signaling.

## Discussion

Corneal transparency is critical for maintenance of vision. Corneal wounds can be treated with topical antibiotics, tear substitutes, and contact lenses; however, at best, many of these traditional treatments only address one aspect of the corneal healing process. Even advanced corneal treatment strategies, such as growth factor derived products or autologous serum, have had limited clinical success, particularly with respect to reducing fibrosis and angiogenesis^29,30^. Here, we provide evidence that MG53 offers dual functionality to improve corneal healing outcomes by improving epithelial viability and reducing the fibrotic stromal response.

We identified native MG53 protein in the tear film, cornea epithelia, and aqueous humor, indicating that MG53 may play a protective role in physiological conditions in the cornea. This finding suggests that topical application of exogenous rhMG53 protein as a potential therapy for corneal wound healing is not likely to cause adverse effects. Autologous serum has been used for treatment of various corneal diseases, and the beneficial effects were largely attributed to growth factors and cytokines^29,30^. While growth factors can promote healing of the corneal epithelium, they may also have side effects. For example, serum TGF-β may promote fibrotic remodeling of the cornea, and cytokines, such as IL-6, IL-1β, and TNF-α, can cause corneal inflammation^29,30^. We have previously demonstrated that MG53 is present in blood serum derived from both rodents and humans^17,19^. In principle, MG53 may serve as one important component of the autologous serum that promotes corneal healing and reduces scarring. One can envision that supplementing exogenous rhMG53 to the autologous serum preparation could offer more beneficial effects for treatment of ocular diseases.

In support of this hypothesis, we showed that rhMG53 can maintain hCEC viability following injury in vitro and improve re-epithelialization of the cornea in a rat model of corneal wound healing. More importantly, we demonstrated that rhMG53 treatment led to reduced corneal stromal fibrosis and vascularization associated with alkaline-induced corneal wound healing.

The cornea is an active tissue that undergoes rapid regeneration and physiological turnover within 10–14 days^31,32^, necessitating an efficient biological mechanism for injury-repair and regeneration. While corneas derived from the *mg53−/−* mice show normal morphology under basal conditions, they had thinner epithelium and developed increased stromal haze with encroachment of vessels into the axial cornea following alkaline injury when compared with the wild type littermates. Such findings are similar to our previous observations with the *mg53−/−* muscle and heart tissues, e.g., both tissues appear to be normal at basal conditions but display pathologies following injury-repair^12,19,33^. Our identification of MG53 as an intrinsic component of corneal protection has important value for the basic physiology of ocular research, as well as potential translational applications in treating corneal injuries. The marked pathological corneal response observed in the wounded *mg53−/−* mice may also be linked to potential defects in corneal stem cell function in the absence of MG53. Investigating the role of MG53 in protection against corneal stem cell injury and maintenance of their renewal capacity will constitute an important area of future research. Moreover, the interaction between corneal epithelial and stromal layers has been shown to play a critical role in corneal wound healing^24,25^. Since our studies demonstrate that MG53 is present in corneal epithelial cells and can be taken up by corneal fibroblasts, it is a logical future research direction to study the potential role of MG53 in modulating the cross-talk between the epithelium and stroma in corneal physiology and disease.

In the present study, we observed that, in addition to its known plasma membrane repair function, rhMG53 can also reduce fibrotic remodeling associated with corneal wound healing in the rat model. While fibrotic remodeling might be a secondary consideration in other diseases, it is a critical problem in the cornea. Fibrotic corneal healing directly results in loss of transparency and normal vision. Compared to other growth factors used for corneal treatment, the anti-fibrotic function of MG53 is unique and advantageous. The ability to reduce fibrosis contributed to the improved corneal transparency following rhMG53 treatment. We have data to show that exogenously applied rhMG53 can enter corneal fibroblasts to alter intracellular TGF-β signaling and suppress fibrotic remodeling associated with corneal injury. Further dissection of the molecular interaction between MG53 and the intracellular factors associated with fibrotic remodeling should provide important clues for developing rhMG53-based treatment strategies for corneal injuries.

Overall, we believe that MG53 has several merits over currently available corneal wound therapies. MG53 is present in the tear film and aqueous humor in multiple species, supporting a potential corneal protective role of MG53 in the physiological condition. Our study revealed a bi-functional role for MG53 in facilitating rapid injury-repair of corneal wounds and reducing fibrotic vascularization associated with corneal injuries. Targeting the dual function of MG53 may offer superior strategy to combat difficult-to-treat corneal wounds.

## Methods

### rhMG53 protein production and quality control

*E. coli* fermentation was used to obtain high quality (>97% purity) rhMG53 protein as described^19^. The membrane protective activity of rhMG53 from each preparation was determined with established micro-glass bead injury assay as described previously^15,19^.

### Cell culture

Human telomerase-immortalized corneal epithelial cells (hCEC; generously provided by Dr. Danielle Robertson, University of Texas Southwestern) were maintained in keratinocyte growth medium (KGM)-2 supplemented with KGM-2 SingleQuot Kit Supplements and Growth Factors (Lonza, Basel, Switzerland), in a 5% CO_2_ humidified incubator at 37 °C, and passaged every 3–5 days.

Primary corneal fibroblasts were prepared from superficial keratectomy samples obtained from the axial cornea of cadaveric canine globes. First, epithelium was mechanically debrided and explants, approximately 5 mm in diameter and 250 µm in depth, comprised of stromal tissue only were place in culture dishes containing maintained in Dulbecco’s modified Eagle’s medium (DMEM) supplemented with 10% fetal bovine serum and 1% penicillin/streptomycin in a 5% CO_2_ incubator. Western blot analysis evaluating vimentin and cytokeratin expression verified the stromal origin of the cells (Supplementary Fig. 1a). We also performed immunofluorescent staining to confirm expression of vimentin in fibroblasts, not in hCEC (Supplementary Fig. 1b).

For treatment with TGF-β and rhMG53, fibroblasts (seeded at 5 × 10^4^ cells per cm^2^) grew to 70% confluence, before being washed twice with serum-free media and subjected to treatment in serum-free DMEM. Cells were treated with DMEM (control), in the presence of either TGF-β (10 ng mL^−1^), rhMG53 (50 μg mL^−1^), or a combination of both TGF-β and rhMG53 for varying times to investigate myofibroblast differentiation.

### Cell scratch wound healing assay

To induce cellular migration, an in vitro scratch test was performed using serum-starved cells; to generate myofibroblasts, fibroblasts were first treated with TGF-β (10 ng mL^−1^). Cells were allowed to grow to 90% confluence and a 1-mm scratch was subsequently made in the cellular monolayer before being treated with 0 or 50 µg mL^−1^ rhMG53. Photomicrographs were taken immediately after the scratch and then every 8 h until restoration of the monolayer. ImageJ software (National Institutes of Health, Bethesda, MD) was used to quantify the change in area over time.

### Confocal live cell imaging

For live cell imaging of MG53-mediated cell membrane repair in hCEC, transfection of GFP-MG53 into hCECs was performed using the Lipofectamine LTX reagent (Life Technologies), per manufacturer’s instructions. hCECs expressing GFP-MG53 were subsequently subjected to microelectrode penetration-induced injury to the plasma membrane as previously described^12^. Cells were imaged using confocal microscopy (Zeiss LSM780). For visualizing the dynamic process of rhMG53 entry into the corneal fibroblasts, rhMG53 and bovine serum albumin (BSA) were labeled with Alexa Fluor^TM^ 647 by Alexa Fluor™ 647 Protein Labeling Kit (Life Technologies, Cat. No. A20173). Labeled rhMG53 or BSA was added to the culture medium of primary corneal fibroblasts and intracellular signal of Alexa 647 was imaged at indicated time points by a confocal microscope. Intracellular fluorescent intensity of Alexa 647 at each time point was quantified by ImageJ software. To visualize cell morphology for fluorescence quantification, the cells were counterstained with MitoTracker Green (Life Technologies, Cat. No. M7514).

### CRISPR/Cas9 mediated MG53 knockout

CRISPR/Cas9 mediated MG53 knockout was performed following our previous publications^34,35^. Briefly, 2 × 10^5^ hCEC cultured in antibiotic-free medium were plated in 6-well plates. The guide RNA probe sequences were obtained from CRISPR design (http://crispr.mit.edu/). Total two guide RNA sequences (5′-AGAACGGTGCCATCCGCCGC-3′ and 5′-CGGGCGCGTCGAACAGCTGC-3′) were tested and the one (5′-AGAACGGTGCCATCCGCCGC-3′) with higher knockout efficiency was used in our experiments. Twenty four hours later, after cells reached 80% confluence, CRISPR/Cas9 MG53 plasmid was transfected into the hCEC using Lipofectamine 3000, according to the manufacturer’s instructions. Forty-eight hours post-transfection, the culture medium was aspirated and replaced with fresh medium containing puromycin (1 μg mL^−1^) to select and establish the stably transfected cells.

### In vitro cell membrane injury assay

In vitro cell membrane injury repair assay was performed as described previously^15,19^. hCECs were suspended in Dulbecco’s PBS at a concentration of 6.0 × 10^5^ cells mL^−1^; 150 μL of this cell suspension (9 × 10^4^ hCECs) was added to each well of a 96-well plate with acid-washed glass micro-beads and the indicated dose of rhMG53 (0–200 µg mL^−1^). To induce cell membrane damage, the plate was shaken at 200 rpm for six minutes. Plates were then centrifuged at 3000×*g* for five minutes and 50 μL supernatant was removed. Lactate dehydrogenase (LDH) activity of the supernatant was determined using a LDH Cytotoxicity Detection Kit (TaKaRa). The LDH values from wells without glass beads (no damage) were used to determine the background activity for each condition and were subtracted from experimental values before comparison.

### Western blot

Protein lysates from indicated tissue and cell sources were separated by SDS-PAGE. Proteins were transferred from gels to PVDF membranes at 4 °C. The blots were washed with PBST (PBS+0.5% Tween-20), blocked with 5% milk in PBST for 2 h, and incubated with indicated primary antibodies overnight at 4 °C under rotation. Secondary antibodies, anti-mouse or anti-rabbit IgG HRP conjugated, were applied at 1:5000 dilution and incubated for approximately 1.5 h with shaking at room temperature. Immunoblots were visualized with an ECL plus kit (Pierce). The antibodies used in this study were as follows: rabbit anti-MG53 antibody was generated by our laboratory and the sensitivity and specificity were confirmed by previous studies from multiple research groups^17,36–38^; anti-p-Smad2 antibody (Cell Signaling Technology, Cat. No. 3108, 1:1000 dilution); anti-Smad2 antibody (Cell Signaling Technology, Cat. No. 5339, 1:1000 dilution); anti-Smad5, (Cell Signaling Technology, Cat. No. 12534, 1:1000 dilution); anti-p-Smad5 antibody (Cell Signaling Technology, Cat. No. 9516, 1:1000 dilution); anti-GAPDH antibody (Cell Signaling Technology, Cat. No. 2118s, 1:5000 dilution); anti-alpha-SMA antibody (Invitrogen, Cat. No.14-9760-82, 1:2000 dilution); and anti-fibronectin antibody (Sigma-Aldrich, Cat. No. F3648, 1:2000 dilution).

Semi-quantitative analysis was performed to quantify expression levels of MG53 in canine aqueous humor and human tear samples. Briefly, 0.4 ng purified rhMG53 protein and 20 μL aqueous humor or tear samples were subjected to Western blot analysis. Western blot bands were quantified using ImageJ software (NIH) and the concentration of MG53 in samples were calculated based on signal ratio between purified rhMG53 protein and average of aqueous humor and tear samples. Raw gel images for all Western blots are shown in Supplementary Fig. 2.

### Quantitative RT-PCR analysis

The expression pattern of α-SMA and fibronectin in treated or untreated canine corneal fibroblasts were examined by quantitative real-time PCR (qRT-PCR) analysis. Total RNAs were extracted by using TRIzol reagent (Invitrogen, CA, Cat. No. 15596026), and genome DNA contamination was eliminated by DNase I (Invitrogen, CA, Cat. No. 18047019), according to the manufacturer’s instructions. One microgram of total RNA was reverse transcribed by cDNA synthesis (Thermo Scientific, Cat. No. 1651) and the products were subjected to quantitative real-time PCR, carried out by SYBR Green Real-Time PCR Mix (Thermo Scientific, Cat. No. A25778) on the DNA Engine LightCycler 480 Instrument II (Roche Molecular Systems, Inc,).The canine gene GAPDH was used as an internal control. The primers used in the assay were: α-SMA forward: 5′-AACACGGCATCATCACCAA-3′, α-SMA reverse: 5′-AGGCGTAGAGGGAAAGCA-3′; fibronectin forward: 5′-CCTCTGACGGCGGAACAAACGACCA-3′, fibronectin reverse: 5′- AGAGGGTCCCACGTTGTACTGCTTG-3′; GAPDH forward: 5′-GTGAAGGTGGAGTGAACGGATTTG-3′, GAPDH reverse: 5′- TTTGATGTTGGCGGGAT-3′. All primer sequences used in this study are presented in Supplementary Table 1.

### Animal care and in vivo corneal wound healing models

All animal care and usage followed NIH guidelines and were in accordance with the ARVO Statement for the Use of Animals in Ophthalmic and Vision Research. Rodent studies received IACUC approval by The Ohio State University. For all corneal wound healing models, injury was induced under anesthesia and all animals received topical antibiotics, and topical and systemic analgesics for at least 72 h for pain management.

To evaluate expression of MG53 in the mediums surrounding the cornea, aqueous humor was collected from normal canine cadaveric globes immediately following enucleation. All samples were immediately frozen at −80 °C until analysis.

Both sexes of *mg53−/−* mice and their wildtype littermates (age of 3–6 months) were generated, bred and genotyped as previously described^12^. Briefly, mouse tail (~3 mm in length) was cut and put into DNA extraction buffer (50 mM NaOH 180 μL). Samples were boiled for 10 min at 95 ℃, then neutralized with 20 µL Tris-HCl buffer (1 M, pH 8.0). Samples were vortexed for 5 s and span down for PCR reaction. The *mg53−/−* mice genotyping PCR primers are: Forward 5′ CCT TCT GCG TCA GGA ACT GTC CTG C 3′, Reverse 5′ CAG CAG TCC CAC CCT GCC TTC ACC G 3′. The PCR reaction sample preparation was as following: 5 μL 2× Fx buffer, 2 μL 2 mM dNTP, 0.25 μL 10 μM primer Forward, 0.25 µL 10 µM primer Reverse, 0.2 µL KOD XtremeTM Hot Start DNA Polymerase (EMD Millipore Corp, Cat: 71975-3), 2 µL dH2O and 0.5 µL mice tail DNA extraction. The PCR reaction program was: 94 ℃ 2 min 1 cycle; 98 ℃ 10 s and 68 ℃ 1 min, 35 cycles; 68 ℃ 2 min, 1 cycle. PCR products were subjected to 1% agarose gel. The knockout band size is 1250 bp and wildtype band is 480 bp. To ensure data reproducibility, mouse tail samples are retained and cataloged for secondary future validation, if necessary. Our *mg53−/−* mouse line has also been validated and used by other laboratories^16,36^. A 2-mm filter paper disc soaked in 1 N NaOH was applied to the axial cornea to induce injury. The clinical opacity and vascularization scores were determined by a masked, board certified veterinary ophthalmologist (AGM), using a modified Hackett-McDonald scoring system. Fourteen days post-alkaline injury, the mice were sacrificed and eyes underwent analyses. In order to obtain details regarding the injury response in *mg53−/−* and wildtype corneas, globes were fixed either for horizontal sectioning or flat mount staining. Analysis of all tissues was performed by an individual masked to the genotype.

For rhMG53 treatment of corneal wounds, Wistar rats (Harlan Laboratories; Indianapolis, IN, both male and female at age of 3–6 month) were used and ophthalmic exams were performed by a masked board certified veterinary ophthalmologist. A wound was introduced by placing a 3-mm piece of filter paper soaked in 1 N NaOH on the axial cornea, thus removing the anterior stroma and overlaying epithelium. Wounded corneas received topical sterile saline with 0 or 100 ng mL^−1^ rhMG53, twice daily for a total of seven days. Size and depth of the corneal wound was verified daily using fluorescein. In conjunction with the use of fluorescein dye to monitor healing rates, the following were clinically evaluated: corneal opacification, vascularization, and cellular infiltrate, conjunctival edema, hyperemia, and discharge, and presence of uveitis. At 7 days following treatment, animals were euthanized and globes were enucleated. In all subsequent histologic analyses, the individual was masked to the treatment.

### Human subjects for tear collection

A total of 6 volunteers were enrolled and only adults 18 years and older were included. Before initiation of the study, this research was approved by The Ohio State University Institutional Review Board. Participants signed an informed consent document following the tenets of the Declaration of Helsinki. Non-reflex tears were collected from the inferior tear prism using 5 µL Drummond glass microcapillary tubes. All samples were immediately frozen at −80 °C until analysis.

### Histopathology and immunohistochemistry

Enucleated globes from mice or rats were fixed in 10% formalin overnight at 4 °C. After fixation, samples were washed three times for 5 min with 70% ethanol. Washed samples were processed and embedded in paraffin. Four μm thick paraffin sections were cut and stained with Hematoxylin-Eosin (H&E). Under light microscopy, the number of epithelial cell layers present was manually counted in four areas within the axial cornea.

Immunofluorescent staining of MG53 was performed using flat mount corneas following a previously published study^23^. Briefly, enucleated globes were fixed in 4% PFA for 1 h at 4 °C. Corneas were carefully dissected from the eye (Leica, Stereo Zoom 4 Microscope) and returned to 4% PFA for fixation overnight at 4 °C. Subsequently, corneas were incubated in blocking buffer (0.5% Triton X-100 and 5% goat serum in PBS) for at least 2 h at room temperature to permeabilize the tissue and prevent nonspecific binding of the primary antibody. Anti-CD31 (BD Biosciences, Cat. No. 550274); and anti-αSMA (Invitrogen, Cat. No.14-9760-82) antibodies in blocking buffer was applied to the tissue and incubated at 4 °C overnight. After six washes (1 h per wash at room temperature) with washing buffer (0.5% Triton X-100 in PBS), a secondary antibody in blocking buffer was applied to the cornea and incubated overnight at 4 °C. Tissues were washed three times with PBS for 1 h each at room temperature. Corneas were transferred into fresh PBS and four incisions from periphery towards the center were made to facilitate imaging.

### Statistical analysis

All data are expressed as mean ± SD. Groups were compared by Student’s *t*-test and analysis of variance for repeated measures. A value of *p* < 0.05 was considered significant.

### Reporting summary

Further information on experimental design is available in the Nature Research Reporting Summary linked to this article.

## Supplementary information

Reporting Summary

Description of Additional Supplementary Files

Supplementary Information

Supplementary Data 1

## Data Availability

The authors declare that the data of this study are available within the article and its supplementary information files.
