# Supplementary material for: MG53 promotes corneal wound healing and mitigates fibrotic remodeling in rodents
Source: Commun Biol. 2019 Feb 20;2:71. doi: 10.1038/s42003-019-0316-7 (PMC6382791; doi:10.1038/s42003-019-0316-7)
Supplement: Supplementary file 1 — Reporting Summary [file 42003_2019_316_MOESM1_ESM.pdf]

nature research

Corresponding author(s): Hua Zhu

Last updated by author(s): Jan 7, 2019

Reporting Summary

Nature Research wishes to improve the reproducibility of the work that we publish. This form provides structure for consistency and transparency in reporting. For further information on Nature Research policies, see Authors & Referees and the Editorial Policy Checklist.

Statistics

For all statistical analyses, confirm that the following items are present in the figure legend, table legend, main text, or Methods section.

n/a	Confirmed	
☐	☒	The exact sample size (n) for each experimental group/condition, given as a discrete number and unit of measurement
☐	☒	A statement on whether measurements were taken from distinct samples or whether the same sample was measured repeatedly
☐	☒	The statistical test(s) used AND whether they are one- or two-sided<br>*Only common tests should be described solely by name; describe more complex techniques in the Methods section.*
☐	☒	A description of all covariates tested
☐	☒	A description of any assumptions or corrections, such as tests of normality and adjustment for multiple comparisons
☐	☒	A full description of the statistical parameters including central tendency (e.g. means) or other basic estimates (e.g. regression coefficient) AND variation (e.g. standard deviation) or associated estimates of uncertainty (e.g. confidence intervals)
☐	☒	For null hypothesis testing, the test statistic (e.g. F, t, r) with confidence intervals, effect sizes, degrees of freedom and P value noted<br>*Give P values as exact values whenever suitable.*
☒	☐	For Bayesian analysis, information on the choice of priors and Markov chain Monte Carlo settings
☒	☐	For hierarchical and complex designs, identification of the appropriate level for tests and full reporting of outcomes
☒	☐	Estimates of effect sizes (e.g. Cohen's d, Pearson's r), indicating how they were calculated

Our web collection on statistics for biologists contains articles on many of the points above.

Software and code

Policy information about availability of computer code

Data collection	ImageJ 1.49m
Data analysis	Microsoft excel 2013; GraphPad Prism 5

For manuscripts utilizing custom algorithms or software that are central to the research but not yet described in published literature, software must be made available to editors/reviewers. We strongly encourage code deposition in a community repository (e.g. GitHub). See the Nature Research guidelines for submitting code & software for further information.

Data

Policy information about availability of data

All manuscripts must include a data availability statement. This statement should provide the following information, where applicable:

- Accession codes, unique identifiers, or web links for publicly available datasets
- A list of figures that have associated raw data
- A description of any restrictions on data availability

The authors declare that all data supporting the findings of this study are available within the paper.

Field-specific reporting

Please select the one below that is the best fit for your research. If you are not sure, read the appropriate sections before making your selection.

☒ Life sciences ☐ Behavioural & social sciences ☐ Ecological, evolutionary & environmental sciences

For a reference copy of the document with all sections, see nature.com/documents/nr-reporting-summary-flat.pdf

Life sciences study design

All studies must disclose on these points even when the disclosure is negative.

Sample size	For in vivo corneal wound healing experiments. We didn't use software to determine the sample size. We chose the sample sizes for each in vivo experiment base on our previous experiences of in vivo corneal wound healing.
Data exclusions	No data were excluded.
Replication	The biochemical and molecular biology experiments in this paper have been repeated at least 2 times (total 3 independent experiments). We didn't notice failure of the replication experiments.
Randomization	For corneal injury experiments on rats and mg53-/- mice, we randomized the rats for different treatments.
Blinding	As for the cornea injury experiments, the operators were blinded with mouse genotypes and treatment reagents. In all subsequent histologic analyses, the individual was masked to the treatment.

Reporting for specific materials, systems and methods

We require information from authors about some types of materials, experimental systems and methods used in many studies. Here, indicate whether each material, system or method listed is relevant to your study. If you are not sure if a list item applies to your research, read the appropriate section before selecting a response.

Materials & experimental systems

n/a	Involved in the study	
☐	☒	Antibodies
☐	☒	Eukaryotic cell lines
☒	☐	Palaeontology
☐	☒	Animals and other organisms
☒	☐	Human research participants
☒	☐	Clinical data

Methods

n/a	Involved in the study	
☒	☐	ChIP-seq
☒	☐	Flow cytometry
☒	☐	MRI-based neuroimaging

Antibodies

Antibodies used	anti-p-Smad2 antibody (Cell Signaling Technology, Cat. No. 3108); anti-Smad2 antibody (Cell Signaling Technology, Cat. No. 5339); anti-Smad5, (Cell Signaling Technology, Cat. No. 12534); anti-p-Smad5 antibody (Cell Signaling Technology, Cat. No. 9516); anti-GAPDH antibody (Cell Signaling Technology, Cat. No. 2118s); anti-alpha-SMA antibody (Invitrogen, Cat. No.14-9760-82); anti-fibronectin antibody (Sigma-Aldrich, Cat. No. F3648); and anti-Vimentin antibody (EMD Millipore, Cat. No. MAB3400)
Validation	We validated the antibodies based on molecular weight, reports on the manufacture websites and published research articles that cited the same source of the antibodies as we used in our study.

Eukaryotic cell lines

Policy information about cell lines

Cell line source(s)	Human telomerase-immortalized corneal epithelial cells (hCEC; generously provided by Dr. Danielle Robertson, University of Texas Southwestern)<br>Primary corneal fibroblasts were prepared from superficial keratectomy samples obtained from the axial cornea of cadaveric canine globes
Authentication	We used cell type specific antibodies to perform Western blot and immunofluorescent staining to authenticate the cell lines. Results are shown in supplemental Figure 1.
Mycoplasma contamination	The cell lines used in this study were not tested for mycoplasma contamination
Commonly misidentified lines<br>(See ICLAC register)	No commonly misidentified lines have been used in this study.

Animals and other organisms

Policy information about studies involving animals; ARRIVE guidelines recommended for reporting animal research

Laboratory animals	Male Wistar rats (Harlan Laboratories) were used for corneal wound healing studies. Male and female mg53-/- mice and their

Laboratory animals	wild type littermates were used for corneal wound healing experiments.
Wild animals	the study did not use wild animals.
Field-collected samples	This study did not use samples collected in the field.
Ethics oversight	The animal protocol used in the study has been approved by IACUC from The Ohio State University.

Note that full information on the approval of the study protocol must also be provided in the manuscript.
