# Supplementary material for: MG53 promotes corneal wound healing and mitigates fibrotic remodeling in rodents
Source: Commun Biol. 2019 Feb 20;2:71. doi: 10.1038/s42003-019-0316-7 (PMC6382791; doi:10.1038/s42003-019-0316-7)
Supplement: Supplementary file 2 — Description of Additional Supplementary Files [file 42003_2019_316_MOESM2_ESM.docx]

**Description of Additional Supplementary Files**

**File name:** Supplementary Data 1

**Description:** Source data underlying the graphs presented in Figures 1f, 2b, 2c, 2e, 2f, 2h, 2j, 3c, 3d, 3f, 3g, 4c, 5b, 5c, 5d, 5e.
