# Supplementary material for: MG53 promotes corneal wound healing and mitigates fibrotic remodeling in rodents
Source: Commun Biol. 2019 Feb 20;2:71. doi: 10.1038/s42003-019-0316-7 (PMC6382791; doi:10.1038/s42003-019-0316-7)
Supplement: Supplementary file 3 — Supplementary Information [file 42003_2019_316_MOESM3_ESM.pdf]

Supplementary Figure 1

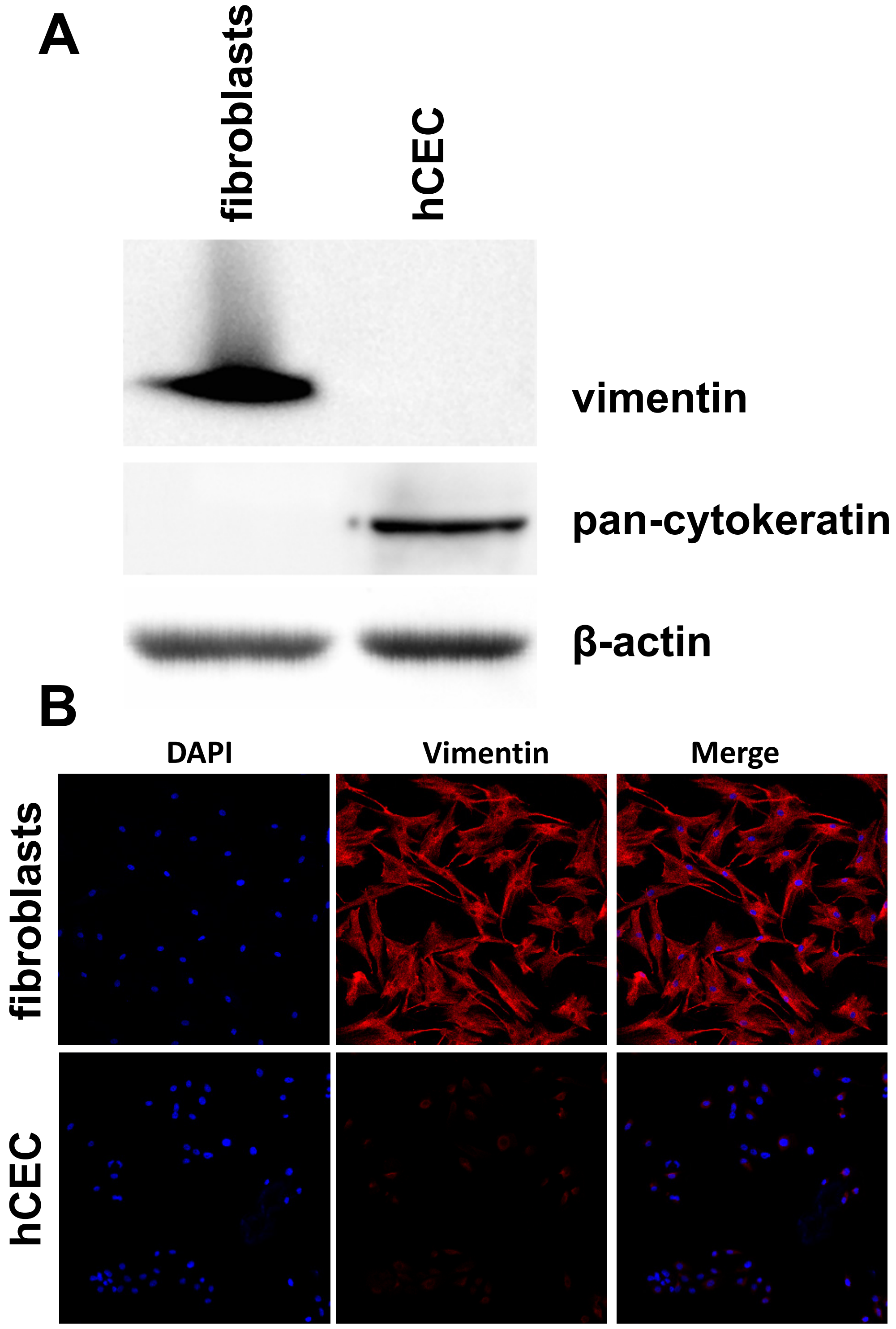

Supplementary Figure 1. Authentication of primary corneal fibroblasts. (**A**) Following isolation of corneal fibroblasts Western blot analysis was performed. Vimentin was used as a marker of fibroblasts and cytokeratin was used as a marker of corneal epithelial cells. The fibroblasts were positive for vimentin and negative for cytokeratin, while human corneal epithelial cells (hCEC) were positive for cytokeratin and negative for vimentin. (**B**) Immunostaining of vimentin was performed. Fibroblasts were positive for vimentin, while hCEC is negative for vimentin.

Supplementary Table 1

The sequences of oligos used in the paper

Name	Sequence
MG53 gRNA-1	5’-AGAACGGTGCCATCCGCCGC-3’
MG53 gRNA-2	5’-CGGGCGCGTCGAACAGCTGC-3’
α-SMA forward primer	5’-AACACGGCATCATCACCAA-3’
α-SMA reverse primer	5’-AGGCGTAGAGGGAAAGCA-3’
fibronectin forward primer	5’-CCTCTGACGGCGGAACAAACGACCA-3’
fibronectin reverse primer	5’-AGAGGGTCCCACGTTGTACTGCTTG-3’
GAPDH forward primer	5’-GTGAAGGTGGAGTGAACGGATTTG-3’
GAPDH reverse primer	5’-TTTGATGTTGGCGGGAT-3’
MG53 genotyping forward primer	5’-CCTTCTGCGTCAGGAACTGTCCTGC-3’
MG53 genotyping reverse primer	5’-CAGCAGTCCCACCCTGCCTTCACCG-3’

Figure 1A

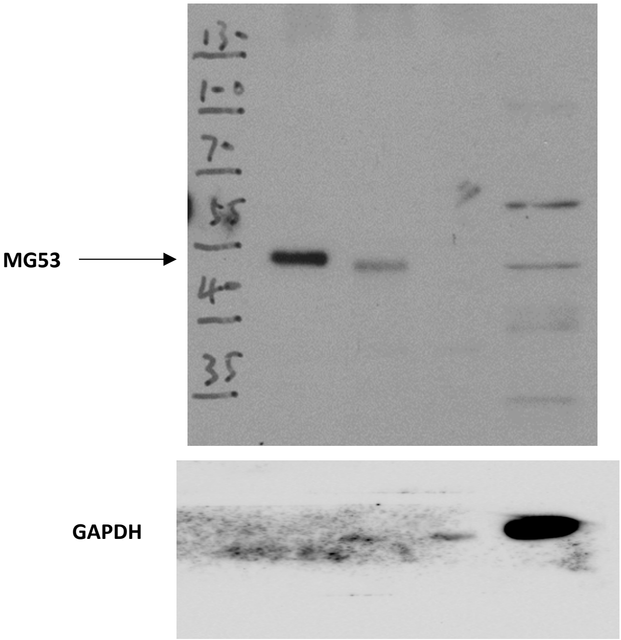

Figure 1B

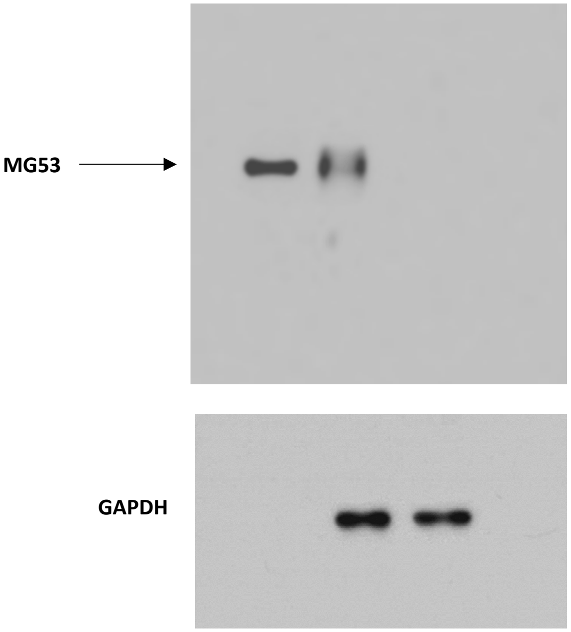

Figure 1C

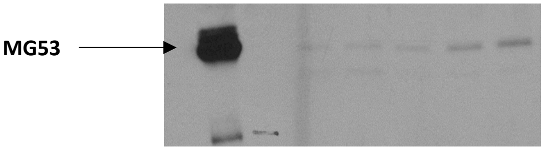

Figure 1D

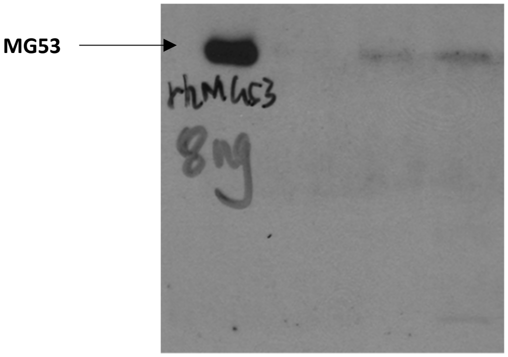

Figure 5C

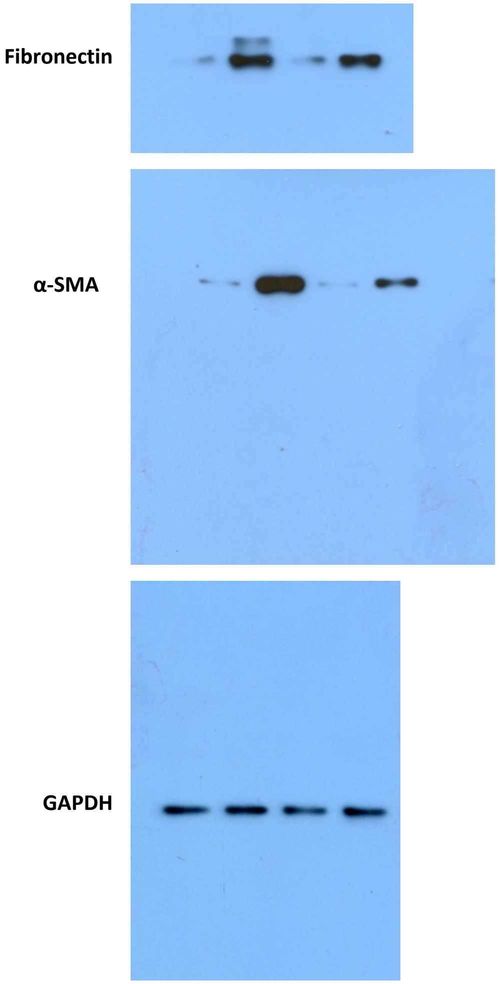

Figure 5E

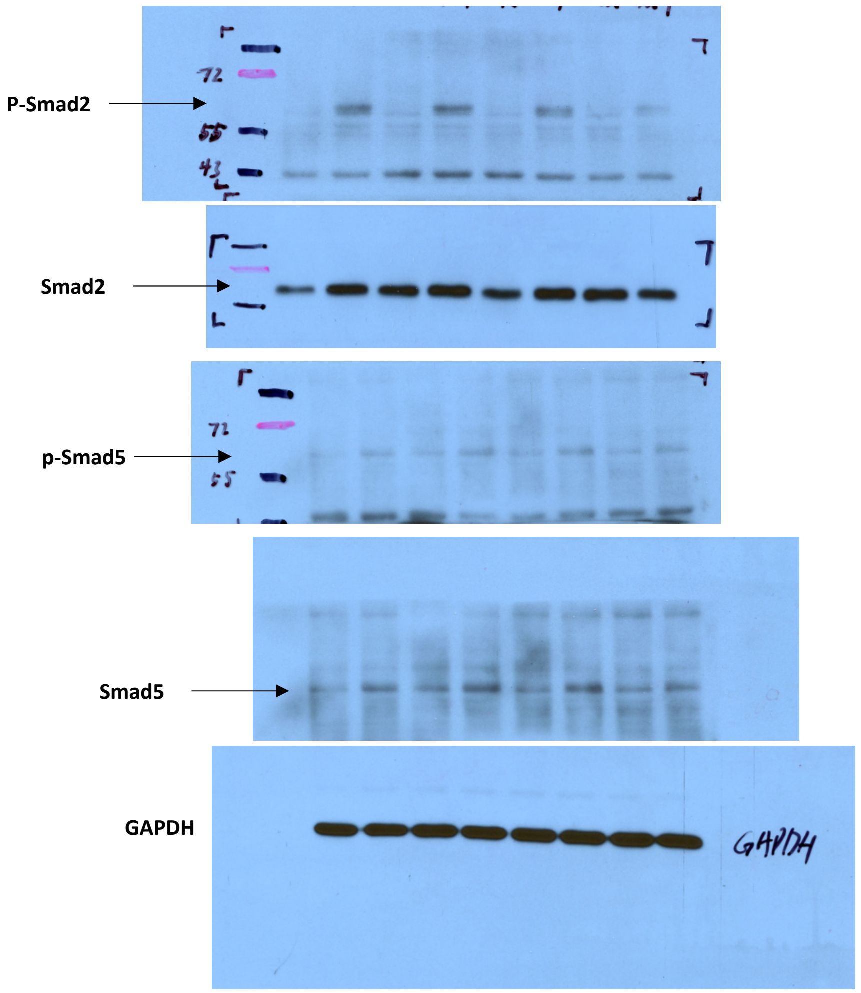

Supplementary Figure 2

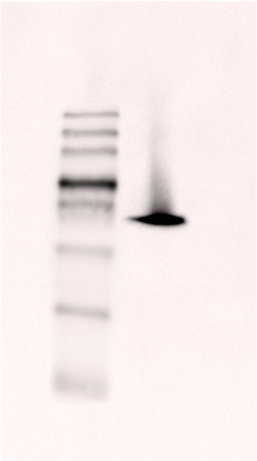

Vimentin

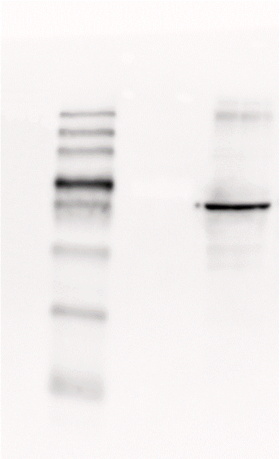

Pan-cytokeratin

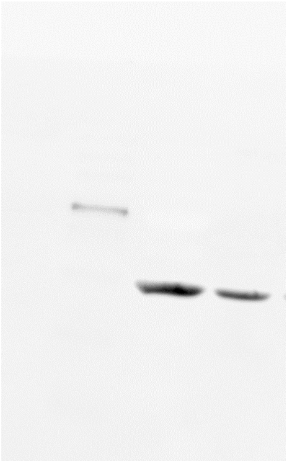

β-actin
